# Supplementary material for: Family’s perceptions of their members who use nyaope in Tshwane, South Africa
Source: PLoS One. 2025 Mar 28;20(3):e0318794. doi: 10.1371/journal.pone.0318794 (PMC11952264; doi:10.1371/journal.pone.0318794)
Supplement: Attachment — Submitted filename: Response_response to reviewers_revised data availability.docx [file pone.0318794.s001.docx]

**Family’s perceptions of their members who use Nyaope in Tshwane, South Africa**

| **Your comments** | **Our answers** | **Changes** |
| --- | --- | --- |
| **Journal requirements:**  When submitting your revision, we need you to address these additional requirements.  1.Please ensure that your manuscript meets PLOS ONE's style requirements, including those for file naming. The PLOS ONE style templates can be found at:  https://journals.plos.org/plosone/s/file?id=wjVg/PLOSOne_formatting_sample_main_body.pdf and  https://journals.plos.org/plosone/s/file?id=ba62/PLOSOne_formatting_sample_title_authors_affiliations.pdf | Accepted |  |
| 2.We note that you have indicated that there are restrictions to data sharing for this study. For studies involving human research participant data or other sensitive data, we encourage authors to share de-identified or anonymized data. However, when data cannot be publicly shared for ethical reasons, we allow authors to make their data sets available upon request. For information on unacceptable data access restrictions, please see http://journals.plos.org/plosone/s/data-availability#loc-unacceptable-data-access-restrictions.  Before we proceed with your manuscript, please address the following prompts:  a) If there are ethical or legal restrictions on sharing a de-identified data set, please explain them in detail (e.g., data contain potentially identifying or sensitive patient information, data are owned by a third-party organization, etc.) and who has imposed them (e.g., a Research Ethics Committee or Institutional Review Board, etc.). Please also provide contact information for a data access committee, ethics committee, or other institutional body to which data requests may be sent.  b) If there are no restrictions, please upload the minimal anonymized data set necessary to replicate your study findings to a stable, public repository and provide us with the relevant URLs, DOIs, or accession numbers. Please see http://www.bmj.com/content/340/bmj.c181.long for guidelines on how to de-identify and prepare clinical data for publication. For a list of recommended repositories, please see https://journals.plos.org/plosone/s/recommended-repositories. You also have the option of uploading the data as Supporting Information files, but we would recommend depositing data directly to a data repository if possible. Please update your Data Availability statement in the submission form accordingly. | Accepted, we will make data to be available on request through the ethic committee.  Accepted | The restriction to data is enforced both directly by the participant consent and indirectly by the Stellenbosch University Human Ethic Committee, which authorized us to conduct the study. Due to the possibility of retaliation from drug (nyaope) dealers in the communities where we collected data, the consent form clearly protected the participant's identity, and participants agreed to participate in the study knowing that the data collected would be protected by a password known only to the researchers, used only for research purposes, and would not be made public. Furthermore, the collected data contained potentially sensitive information and highlighted several legal gaps, exposing officials. Much like the participants' perception that certain police personnel collaborated with nyaope suppliers.  To comply with the journal's policy and prevent breaching confidentiality, would you mind if the data is accessible through Human Research Ethic Committee of Stellenbosch University who authorize this study?  For data access, please contact Charmaine Khumalo, Head of Health Research Ethics, at 021 938 9075; ckhumalo@sun.ac.za.  Or contact Mrs. Brightness Nxumalo, Coordinator, Health Research Ethics Committee 2 (HREC 2), at 021 938 9207; brightness@sun.ac.za.    **Please amend our Data Availability declaration on our behalf to include the details we have provided.**  Because the information was about drugs and potential retaliation, the participants did not authorize for it to be available, thus we only provided it upon request from the ethics committee |
| 3. Please review your reference list to ensure that it is complete and correct. If you have cited papers that have been retracted, please include the rationale for doing so in the manuscript text, or remove these references and replace them with relevant current references. Any changes to the reference list should be mentioned in the rebuttal letter that accompanies your revised manuscript. If you need to cite a retracted article, indicate the article’s retracted status in the References list and also include a citation and full reference for the retraction notice.  [Note: HTML markup is below. Please do not edit.] | Accepted | We reviewed our reference list to ensure that it was correct, and we added DOIs for those that were available. We included a link in the attached list for those lacking a DOI. None of those cited have been retracted. |
| **Reviewers' comments:**  **Reviewer's Responses to Questions**  Comments to the Author  1. Is the manuscript technically sound, and do the data support the conclusions?  The manuscript must describe a technically sound piece of scientific research with data that supports the conclusions. Experiments must have been conducted rigorously, with appropriate controls, replication, and sample sizes. The conclusions must be drawn appropriately based on the data presented.  Reviewer #1: Yes  Reviewer #2: Yes | Thank you  Thank you |  |
| 2. Has the statistical analysis been performed appropriately and rigorously?  Reviewer #1: N/A  Reviewer #2: Yes | Thank you  Thank you |  |
| 3. Have the authors made all data underlying the findings in their manuscript fully available?  The PLOS Data policy requires authors to make all data underlying the findings described in their manuscript fully available without restriction, with rare exception (please refer to the Data Availability Statement in the manuscript PDF file). The data should be provided as part of the manuscript or its supporting information, or deposited to a public repository. For example, in addition to summary statistics, the data points behind means, medians and variance measures should be available. If there are restrictions on publicly sharing data—e.g. participant privacy or use of data from a third party—those must be specified.  Reviewer #1: No  Reviewer #2: No | Accepted | Because the data concerns drugs and potentially retaliation, the participants did not consent to its release. As a result, data are available upon request through the ethics committee. |
| 4. Is the manuscript presented in an intelligible fashion and written in standard English?  PLOS ONE does not copyedit accepted manuscripts, so the language in submitted articles must be clear, correct, and unambiguous. Any typographical or grammatical errors should be corrected at revision, so please note any specific errors here.  Reviewer #1: Yes  Reviewer #2: Yes | Thank you  Thank you |  |
| **5. Review Comments to the Author**  Please use the space provided to explain your answers to the questions above. You may also include additional comments for the author, including concerns about dual publication, research ethics, or publication ethics. (Please upload your review as an attachment if it exceeds 20,000 characters)  Reviewer #1: The minor issues that need attention are the following:  Failure to avail data has been explained  line 28: it should be nyaope use.....  lines 60 to 64: the authors must also indicate that even the composition differs across samples because not all samples have milk, or paracetamol, etc. there are publications for such information  Nyaope is not a proper noun and should not be written in capital N, except at the beginning of a sentence  lines 130 to 131. it is not clear what linguistic discrimination refers to  line 452: the authors must explain what too late means | Thank you  Accepted  Accepted  Accepted  Accepted  Accepted  Accepted | Correction made:… , nyaope use has evolved… line 28  11. Article added: Khine AA, Mokwena KE, Huma M, Fernandes L. Identifying the composition of street drug nyaope using two different mass spectrometer methods. African Journal of Drug & Alcohol Studies. 2015;14(1).  Corrections made throughout  Tshwane is home to a diverse population that speaks a variety of languages (Afrikaans, English, Si Tswana, Si Pedi, Si Zulu). Participants were free to utilize the language of their choice, as the interviewer was fluent in all of them. Line 130-132  Most of the relatives became aware of the problem only once it was too late as the users were already showing signs of dependency on nyaope. Line 453-454 |
| Reviewer #2: General comments  I very much enjoyed reading the manuscript. It is an interesting study of public health concern and deserves a scientific merit. The manuscript followed the structure of a journal article. The findings were arrived at through appropriate qualitative processes and analysis.  However, the authors stated that “data cannot be shared publicly because of participants' privacy”. If necessary, the authors can still share the data with PLOS ONE in a more anonymized way. The authors can remove personal identifiers from the transcripts and that may help. If by any reason this is not possible, then it should be stated. For instance, participants during consenting may have been informed that the data (whether anonymized or not) would not be shared with any third party. If so, then it should be clearly stated as a reason for not sharing the data.  Below are some few minor comments for consideration.  The manuscript is well-written so I do not have much comments except for a few minor ones as below.  Data analysis  • The statement “The authors used the service of a professional linguist to translate the transcribed scripts from the vernacular languages into English and to transcribe the interviews verbatim” is not clear. Did the authors first have vernacular transcripts and then these transcripts translated into English? Or was the translation done while listening to the vernacular audios and transcribing straight ahead to English?  Ethical considerations  • Since the topic is sensitive, it would be interesting to demonstrate how Informed Consent was obtained. This could be in a brief sentence. For instance, having such an interview on such a topic could provoke emotions. This is a risk that should be explained to the participant when obtaining informed consent.  Results  • Sentence correction in line 203: Take off the first “are” in the sentence “…I would say that people who are using are Nyaope are dissimulators….”.  Strengths and limitations of the study  • This is a sensitive topic since it is about SUD. Couldn’t it have been better to use individual In-Depth Interviews rather than group interviews? Some persons might not bring out certain sensitive information in a group discussion. I think this could be a weakness/limitation of the study that is worth mentioning. | Thank you  Accepted  Accepted  Accepted  Accepted  Accepted | Because the data involves drugs and the possibility of retaliation, the participants did not consent to its distribution. As a result, we have considered only granted access to the data when requested through the ethics committee.  The authors used the service of a professional linguist who translated vernacular audios and transcribing straight ahead to English. Line 154-155    Indeed, we took some precautions: Because the subject matter is sensitive, conducting such an interview may elicit strong emotions. We mentioned to the participants that they might withdraw at any point of the interview or even after the interview if they felt like it, and that the social workers at COSUP were available for debriefing, if necessary, after the interview. Line 179-182  “…I would say that people who are using nyaope are dissimulators….”. line 208  In fact, it is a limitation. One of our recommendations was to undertake additional studies. This study is part of a larger research endeavor. We conducted detailed interviews with users. |
| 6. PLOS authors have the option to publish the peer review history of their article (what does this mean?). If published, this will include your full peer review and any attached files.  If you choose “no”, your identity will remain anonymous but your review may still be made public.  Do you want your identity to be public for this peer review? For information about this choice, including consent withdrawal, please see our Privacy Policy.  Reviewer #1: No  Reviewer #2: Yes: Aaron Kampim |  |  |

Reference list check

| **References** |  |
| --- | --- |
| 1. Nzaumvila DK, Helliwell T, Mash R. Perspectives of community members on the factors underlying the use of and dependency to Nyaope in Tshwane: A descriptive exploratory qualitative study. S Afr Fam Pract. 2023;65(1):a5751. | doi.org/ 10.4102/safp.v65i1.5715 |
| 2. Mokwena KE, Huma M. Experiences of Nyaope users in three provinces of South Africa: Substance abuse. Afr. J. Phys. Health Educ. Recreat. Danc. 2014;20(Suppl. 1):352–63. | doi/epdf/10.10520/EJC162264 |
| 3. Varshney K, Browning SD, Debnath SK, Shet P, Shet D. A systematic review of risk factors and consequences of Nyaope usage: The illicit street drug containing HIV antiretrovirals. AIDS Behav. 2022 Jul 27; Epub ahead of print. doi: 10.1007/s10461-022-03791-6 | doi: 10.1007/s10461-022-03791-6 |
| 4. Mokwena K. “Consider our plight”: A cry for help from Nyaope users. Health SA Gesondheid [online]. 2016;21(1):137–42. doi: 10.1016/j.hsag.2015.09.001 | DOI: 10.1016/j.hsag.2015.09.001 |
| 5. Madiga MC, Mokwena K. Depression symptoms among family members of Nyaope users in the City of Tshwane, South Africa. Int J Environ Res Public Health. 2022 Mar 30;19(7):4097. doi: 10.3390/ijerph19074097 | Doi: 10.3390/ijerph19074097. PMID: 35409779 |
| 6. Mokwena K. Social and public health implications of the legalisation of recreational cannabis: A literature review. Afr J Prm Health Care Fam Med. 2019;11(1):a2136. doi: 10.4102/phcfm.v11i1.2136 | doi: [10.4102/phcfm.v11i1.2136](https://doi.org/10.4102%2Fphcfm.v11i1.2136) |
| 7. Ndlovu NA, Morgan N, Malapile S, Subramaney U, Daniels W, Naidoo J, et al. Fronto-temporal cortical atrophy in 'Nyaope' combination heroin and cannabis use disorder. Drug Alcohol Depend. 2021;221:108630. doi: 10.1016/j.drugalcdep.2021.108630 | doi: 10.1016/j.drugalcdep.2021.108630 |
| 8. Harker N, Lucas WC, Laubscher R, Dada S, Myers B, Parry CD. Is South Africa being spared the global opioid crisis? A review of trends in drug treatment demand for heroin, Nyaope and codeine-related medicines in South Africa (2012–2017). Int J Drug Policy. 2020;83:102839. doi: 10.1016/j.drugpo.2020.102839 | Doi: 10.1016/j.drugpo.2020.102839 |
| 9. Mokwena K. The novel psychoactive substance ‘Nyaope’ brings unique challenges to mental health services in South Africa. Int Emer Ment Health and Human Res. 2015;17(1):251–2. | https://www.omicsonline.org/open-access/the-novel-psychoactive-substance-nyaope-brings-unique-challenges-to-mental-health-services-in-south-africa-1522-4821-17-152.pdf |
| 10. Mthembi PM, Mwenesongole EM, Cole MD. A validated method for the analysis and profiling of ‘Nyaope’ using gas chromatography: Mass spectrometry. S Afr J Sci. 2021; 117(11/12):Art. #8738. doi: 10.17159/sajs.2021/8738 | doi: 10.17159/sajs.2021/8738 |
| 11. Khine AA, Mokwena KE, Huma M, Fernandes L. Identifying the composition of street drug nyaope using two different mass spectrometer methods. African Journal of Drug & Alcohol Studies. 2015;14(1). | https://www.ajol.info/index.php/ajdas/article/view/131003 |
| 12. Khine AA, Mokwena KE. Drug interactions in the constituents of street drug mixture ‘Nyaope’ in South Africa: A mini-review. Afric J Drug Alcohol Stud. 2016;15(2):92–101. | ajol-file-journals_391_articles_156989_submission_proof_156989-4657-409198-1-10-20170602.pdf  1 |
| 13. Mphahlele LS, Setwaba M, Nel K, Govender I. Experience and behaviour(s) of students who use Nyaope drug at a South African university. Open Public Health J. 2022;(15):e187494452212061. doi: 10.2174/18749445-v15-e221206-2022-94 | doi: 10.2174/18749445-v15-e221206-2022-94 |
| 14. Lefoka MH, Netangaheni TR. A plea of those who are affected most by HIV: The utterances by women who inject Nyaope residing in the City of Tshwane Municipality, Gauteng. Afr J Prm Health Care Fam Med. 2021;13(1):a2416. doi: 10.4102/phcfm.v13i1.2416 | doi: 10.4102/phcfm.v13i1.2416 |
| 15. Benishek LA, Kirby KC, Dugosh KL. Prevalence and frequency of problems of concerned family members with a substanceusing loved one. Am. J. Drug Alcohol Abus. 2011;37:82–8. | https://www.ncbi.nlm.nih.gov/pmc/articles/PMC3073130/ |
| 16. Usher K, Jackson D, O’Brien L. Shattered dreams: Parental experiences of adolescent substance abuse. Int. J. Ment. Health Nurs. 2007;16:422–30. DOI: 10.1111/j.1447-0349.2007.00497.x 17. | DOI: 10.1111/j.1447-0349.2007.00497.x |
| 18. Zauszniewski JA, Bekhet AK. Factors associated with the emotional distress of women family members of adults with serious mental illness. Arch. Psychiatr. Nurs. 2014;28:102–7. DOI: 10.1016/j.apnu.2013.11.003 | DOI: 10.1016/j.apnu.2013.11.003 |
| 19. Watt MH, Meade CS, Kimani S, MacFarlane JC, Choi KW, Skinner D, Sikkema KJ. The impact of methamphetamine (“tik”) on a peri-urban community in Cape Town, South Africa. Int. J. Drug Policy 2014;25:219–25. PMID: 24673783 DOI: 10.1016/j.apnu.2013.11.003 | PMID: 24673783 DOI: 10.1016/j.apnu.2013.11.003 |
| 20. Masombuka J, Mathibela F. Experiences and coping strategies of parents living with adolescents misusing Nyaope substance. Health SA Gesondheid. 2022;27(0):a2042. doi: 10.4102/hsag.v27i0.2042 | doi: 10.4102/hsag.v27i0.2042 |
| 21. Masombuka J, Qalinge L. Outcry and call for relief: Experiences and support needs of parents with Nyaope users. Soc Work. 2020;56(1):51–62. doi: 10.15270/52-2-789 | doi: 10.15270/52-2-789 |
| 22. Hugo J, Shelly S, Llla S, et al. Harm reduction in practice: The community oriented substance use programme in Tshwane. Afr J Prm Health Care Fam Med. 2020;12(1):a2285. doi: 10.4102/phcfm.v12i1.2285 | doi: 10.4102/phcfm.v12i1.2285 |
| 23. Goeieman DS, Nonyane DS, Nzaumvila DK, van Rensburg MNS. Retention of service users on opioid substitution therapy in the City of Tshwane, South Africa. Afr J Prm Health Care Fam Med. 2023;15(1):a3392. doi: 10.4102/phcfm.v15i1.3392 | doi: 10.4102/phcfm.v15i1.3392 |
| 24. Lefoka MH, Netangaheni RT. Factors associated with smoking and transitioning to Nyaope injection amongst women in the City of Tshwane Municipality: A self-report by women. Health SA Gesondheid. 2022;54(3). Health SA Gesondheid. 27(0):a1775. doi: 10.4102/hsag.v27i0.1775 | doi: 10.4102/hsag.v27i0.1775 |
| 25. Mokwena K, Shandukani F, Fernandes L. A profile of substance abuse clients admitted to an in-patient treatment centre in Tshwane, South Africa [Internet]; 2023 [cited 2022 March 5]. Available from: https://purerims.smu.ac.za/en/publications/a-profile-of-substance-abuse-clients-admitted-to-an-in-patient-tr | https://purerims.smu.ac.za/en/publications/a-profile-of-substance-abuse-clients-admitted-to-an-in-patient-tr |
| 26. South African History Online. Pretoria (Tswane): The naming of a city [homepage on the Internet]. [updated 2016 April 16; cited 2019 March 8]. Available from: https://www.sahistory.org.za/article/pretoria-tshwane-naming-city | https://www.sahistory.org.za/article/pretoria-tshwane-naming-city |
| 27. South Africa. Department of Social Development. National drug master plan 2013 – 2017. No date [cited 2023 December 4]. Available from: <https://extranet.who.int/ncdccs/Data/ZAF_B10_National%20drug%20masterplan.pdf> | https://extranet.who.int/ncdccs/Data/ZAF_B10_National%20drug%20masterplan.pdf |
| 28. Bronfenbrenner U. Ecological models of human development. In: Gauvain M, Cole M, editors. Readings on the development of children, 2nd edition. New York: Freeman, 1993; p. 37–43. | chrome-extension://efaidnbmnnnibpcajpcglclefindmkaj/https://www.ncj.nl/wp-content/uploads/media-import/docs/6a45c1a4-82ad-4f69-957e-1c76966678e2.pdf |
| 29. Business Research Methodology. Purposive sampling [homepage on the Internet]. [updated 2019 October 2; cited 2024 September 11]. Available from: <https://research-methodology.net/sampling-in-primary-data-collection/purposive-sampling/> | <https://research-methodology.net/sampling-in-primary-data-collection/purposive-sampling/> |
| 30. Mabuza LH, Govender I, Ogunbanjo GA, Mash B. African primary care research: Qualitative data analysis and writing results. Afr J Prm Health Care Fam Med. 2014;6(1):1–5. doi: 10.4102/phcfm.v6i1.640 | doi: 10.4102/phcfm.v6i1.640 |
| 31. ATLAS.ti Scientific Software Development GmbH (2023) ATLAS.ti Mac (Version 23.2.1) [computer program]. 2023. Available at: https://atlasti.com (downloaded: 2023 July 27). | https://atlasti.com |
| 32. Slep AMS, Heyman RE, Lorber MF, Baucom KJW, Linkh DJ. Evaluating the effectiveness of NORTH STAR: A community-based framework to reduce adult substance misuse, intimate partner violence, child abuse, suicidality, and cumulative risk. Prev Sci. 2020 Oct;21(7):949–59. doi: 10.1007/s11121-020-01156-w. | doi: 10.1007/s11121-020-01156-w |
| 33. Muthelo L, Mbombi MO, Mphekgwana P, Mabila LN, Dhau I, Tlouyamma J, et al. Exploring roles of stakeholders in combating substance abuse in the DIMAMO Surveillance Site, South Africa. Subst Abuse. 2023;17:11782218221147498. doi: 10.1177/11782218221147498 | doi: 10.1177/11782218221147498 |
