# Supplementary material for: Family’s perceptions of their members who use nyaope in Tshwane, South Africa
Source: PLoS One. 2025 Mar 28;20(3):e0318794. doi: 10.1371/journal.pone.0318794 (PMC11952264; doi:10.1371/journal.pone.0318794)
Supplement: Attachment — Submitted filename: Response to editor.docx [file pone.0318794.s002.docx]

**Family’s perceptions of their members who use nyaope in Tshwane, South Africa**

Thank you for the comment.

The table below shows the results of your recommendations. Current changes are highlighted in red in the manuscript with track changes.

| **Your comments** | **Our comment** | **Changes** |
| --- | --- | --- |
| **Add the following headings prior to the references:**  1. Conflict of interest | Accepted | Page 22 (556-559) |
| 2. Funding information | Accepted | Page 22(560-562) |
| 3. Author contribution | Accepted | Page 22(563-571) |
| 4. Acknowledgements | Accepted | Page 22(572-579) |
| 5. Data availability | Accepted | Page 23(581-602) |

Regards

Dr Nzaumvila
